# The Mechanism of Emodin Against Methicillin-Resistant *Staphylococcus aureus* Infection and Research on Synergistic Antibiotics

**DOI:** 10.3390/life15121920

**Published:** 2025-12-15

**Authors:** Chenliang Chu, Liang Qin, Huayong Peng, Tao Kuang, Yongshi Li, Xin Wang, Fenglan Liang, Ping Gao, Xiaoxiong Wang, Deyun Ma

**Affiliations:** 1 School of Food and Pharmaceutical Engineering, Zhaoqing University, Zhaoqing 526061, China; 2 School of Pharmaceutical Sciences, Sun Yat-sen University, Guangzhou 511400, China; 3 College of Life Science, Zhaoqing University, Zhaoqing 526061, China; 4 Zhangjiang Institute for Food and Drug Control, Zhanjiang 524008, China; 5 School of Materials and Environmental Engineering, Shenzhen Polytechnic University, Shenzhen 518055, China

**Keywords:** emodin, methicillin-resistant *Staphylococcus aureus* (MRSA), synergy, β-lactam antibiotics, multi-target mechanism

## Abstract

Methicillin-resistant *Staphylococcus aureus* (MRSA) poses a significant clinical challenge due to its multidrug resistance, particularly to β-lactam antibiotics. This study comprehensively evaluated the natural compound emodin for its anti-*MRSA* activity, mechanisms of action, and potential for synergy with β-lactam antibiotics. Our findings demonstrate that emodin dose-dependently inhibits *MRSA* growth and abrogates biofilm formation at 2× MIC. Mechanistic studies revealed that emodin compromises cell membrane and wall integrity, induces oxidative stress, and downregulates the virulence factors SPA and EsxA. Furthermore, emodin acted synergistically with β-lactam antibiotics: it enhanced the ability of cefalexin to block bacterial adhesion and invasion of HaCat cells, and potentiated the efficacy of amoxicillin in clearing *MRSA* from infected macrophages. In conclusion, emodin employs a multi-target mechanism against *MRSA* and can resensitize the bacterium to conventional β-lactam antibiotics, presenting a promising strategy for combination therapy that may help curb antibiotic use and resistance development.

## 1. Introduction

The escalating dissemination of multidrug-resistant bacteria poses a critical threat to global public health [1,2]. Recent surveillance data from CHINET (2023) highlight the persistent challenge of Methicillin-resistant *Staphylococcus aureus* (MRSA) in China, with its detection rate rising from 28.7% in 2022 to 29.6% [3]. This solidifies MRSA’s status as a leading cause of both community and healthcare-associated infections [4]. Recognized as a highly contagious pathogen, MRSA has been categorized as a serious threat by the U.S. Centers for Disease Control and Prevention [5]. The relentless spread of antimicrobial resistance contributes to increased mortality and substantial economic burdens, underscoring the urgent need for novel therapeutic strategies [6].

Natural products derived from Traditional Chinese medicine (TCM) represent a promising source for combating drug resistance, owing to their structural diversity and capacity for multi-target mechanisms of action [7,8]. *Rheum officinale* Baill, a traditional Chinese medicinal herb, derives its antibacterial activity primarily from anthraquinone constituents, notably its representative compound, emodin [9]. Emodin is a natural hydroxyanthraquinone derivative. It is not only found in rhubarb (primarily in the rhizomes of *Reynoutria japonica* Houtt.) but is also widely present in other medicinal plants such as Polygonum cuspidatum (giant knotweed). With a history of thousands of years in traditional medicine, emodin has been the subject of modern pharmacological research, which has revealed its multiple biological activities, including anti-inflammatory, anti-tumor, and broad-spectrum antibacterial effects [10,11]. Previous research indicates that emodin can increase bacterial membrane permeability and exert anti-biofilm effects [12,13]. A light-dependent, reactive oxygen species (ROS)-mediated antibacterial mechanism has also been suggested for this compound [14]. Furthermore, other rhubarb constituents, such as rhein, have been reported to inhibit MRSA growth via membrane disruption [15]. Nevertheless, the action mechanism of emodin against MRSA and its synergistic potential with conventional antibiotics have not been fully elucidated.

Therefore, this study aims to systematically elucidate the anti-MRSA activity of emodin and its underlying mechanisms. A particular focus will be placed on evaluating its synergistic potential with β-lactam antibiotics. By delineating emodin’s multi-target mechanisms and its role in resensitizing MRSA to β-lactams, this work seeks to provide a scientific foundation for developing novel combination therapies against drug-resistant bacterial infections.

## 2. Materials and Methods

### 2.1. Reagents and Strains

#### 2.1.1. *Rheum officinale* Baill (Part(s) Used: Root and Rhizome.)

In September 2023, rhubarb was obtained from Bangjian Chain Pharmacy in Zhaoqing, Guangdong Province. The *R. officinale* was crushed and filtered through a 50-mesh sieve. The powdered sample was then placed in a self-sealing bag and stored in a desiccator. A voucher specimen of the *R. officinale* (No. A20230910) is preserved in the Pharmacognosy Herbarium of Sun Yat-sen University. The representative active constituent of the *R. officinale* is emodin (C_15_H_10_O_5_), which constitutes the focus of our research.


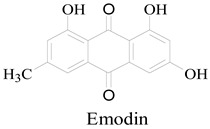


#### 2.1.2. Bacterial Strain

Strain Source: Methicillin-resistant *Staphylococcus aureus* (MRSA, ATCC 43300) was purchased from the Guangzhou Microbial Culture Collection Center, Guangdong Province.

Culture Medium: Mueller Hinton Broth (MHB) was used as the culture medium, which is the standard medium for antibacterial susceptibility testing.

Strain Preservation: MRSA strains were preserved at −80 °C in Mueller-Hinton broth (MHB) supplemented with 20% glycerol. Glycerol is a standard cryoprotectant that prevents bacterial cell damage during low-temperature storage; pure MHB cannot achieve long-term preservation.

Growth Monitoring: Bacterial growth was measured by monitoring the optical density (OD) value using a UV-spectrophotometer

Bacterial Preparation: MRSA in the logarithmic growth phase (typically with an OD_600_ of 0.5–0.6) was collected and used in subsequent experiments to ensure consistent bacterial activity.

#### 2.1.3. Antibiotics

All antibiotics used in the experiment (including cephalexin, amoxicillin, clindamycin, and gentamicin mentioned in subsequent sections) were purchased from Dashenlin Pharmacy.

#### 2.1.4. The Main Detection Kits Used in the Experiment

*Staphylococcus aureus* Protein A (SPA) ELISA Detection Kit (Shanghai Jianglai Biotechnology Co., Ltd. Shanghai, China), *Staphylococcus aureus* EsxA Protein (EsxA) ELISA Kit (Suzhou Xinsaimei Biotechnology Co., Ltd. Suzhou, China), The Chemiluminescence ATP Content Detection Kit (Beyotime Biotechnology Co., Ltd. Shanghai, China),Reduced Glutathione Detection Kit (Solarbio Science & Technology Co., Ltd. Beijing, China),the SOD Detection Kit (Shanghai Bebio Biotechnology Co., Ltd. Shanghai, China), the Prx ELISA Kit (Suzhou Xinsaimei Biotechnology Co., Ltd. Suzhou, China), γ-Glutamylcysteine Ligase (GCL) Kit (Solarbio Science & Technology Co., Ltd. Beijing, China), DCFH-DA ROS fluorescence probe (Solarbio Science & Technology Co., Ltd. Beijing, China), CFDA-SE Fluorescent Probe (Beyotime Biotechnology Co., Ltd. Shanghai, China), Bacterial Protein Extraction Kit (Shanghai Bebio Biotechnology Co., Ltd. Shanghai, China).

### 2.2. Anti-MRSA Activity of Four Major Anthraquinones from the R. officinale

Four major anthraquinone compounds—rhein, emodin, aloe-emodin, and chrysophanol—were isolated from *R. officinale* using established extraction and separation protocols [16]. These compounds were selected for investigation based on their documented status as principal bioactive constituents of this medicinal plant [17]. Additionally, berberine (Aladdin, Cat. No. B414323), flavonols (Aladdin, Cat. No. D650271), and baicalin (Aladdin, Cat. No. B110211) were selected as the ‘classical antibacterial constituents from traditional Chinese medicine’ to serve as a reference system for activity comparison.

The antibacterial activities of these four monomeric compounds against MRSA were evaluated by determining their minimum inhibitory concentrations (MICs) using the broth microdilution method in accordance with Clinical and Laboratory Standards Institute (CLSI) guidelines. Stock solutions of each compound were prepared in ethanol and subsequently diluted in cation-adjusted Mueller-Hinton broth (CAMHB) to achieve the required working concentrations. The final concentration of ethanol in all test wells, including the vehicle control, did not exceed 0.1% (*v*/*v*), which was confirmed to have no effect on bacterial growth [18].

Briefly, a bacterial suspension of MRSA was adjusted to approximately 2 × 10^5^ CFU/mL in CAMHB. A 50 μL aliquot of this suspension was inoculated into each well of a 96-well plate containing 50 μL of serial two-fold dilutions of the test compounds. The concentration ranges tested were 55–1760 μg/mL for emodin, and comparable ranges were used for rhein, aloe-emodin, and chrysophanol. Vancomycin (0.5–8 μg/mL) served as the positive control. The plate was incubated at 37 °C for 24 h. The MIC was defined as the lowest concentration of the compound that completely inhibited visible bacterial growth [18].

### 2.3. Antimicrobial Susceptibility Testing

The antimicrobial susceptibility of MRSA to various antibiotics and the active components from *R. officinale* was evaluated using the E-test (epsilometer test) method, an antibiotic gradient diffusion technique [19]. This quantitative method was employed to determine the minimum inhibitory concentrations (MICs) and identify the most potent anti-MRSA compounds for subsequent mechanistic studies.

Bacterial suspensions were prepared from fresh overnight cultures and adjusted to a turbidity of 0.5 McFarland standard (approximately 1–2 × 10^8^ CFU/mL) in sterile saline. The suspensions were uniformly swabbed onto the surface of Mueller-Hinton agar plates. E-test strips containing predefined antibiotic gradients were then aseptically applied to the inoculated agar surfaces. The tested agents included conventional antibiotics (amoxicillin, cephalexin, gentamicin, and clarithromycin) and the isolated anthraquinones (rhein, emodin, aloe-emodin, and chrysophanol). Plates were incubated at 37 °C for 24 h.

After incubation, the MIC values were read at the intersection of the elliptical zone of inhibition with the E-test strip. Interpretation of susceptibility (susceptible, intermediate, or resistant) for conventional antibiotics followed the breakpoints established by the Clinical and Laboratory Standards Institute (CLSI).

### 2.4. Bacterial Growth Curve Analysis

The effect of emodin on MRSA growth kinetics was determined by time-kill curve analysis. Briefly, logarithmic-phase MRSA cultures were diluted in fresh Mueller-Hinton Broth (MHB) to a density of approximately 1 × 10^6^ CFU/mL. The bacterial suspensions were then treated with emodin at sub-inhibitory (½×MIC) and inhibitory (1×MIC) concentrations. A suspension containing 0.1% (*v*/*v*) ethanol served as the vehicle control. All cultures were incubated at 37 °C with constant shaking at 150 rpm. Bacterial growth was monitored by measuring the optical density at 600 nm (OD_600_) at designated time intervals (0, 5, 10, 15, 20, and 25 h) using a spectrophotometer. Growth curves were plotted as OD_600_ versus time to visualize the bacteriostatic or bactericidal effect of emodin [18].

### 2.5. Crystal Violet Staining Assay for MRSA Biofilm Formation Inhibition

The experiment for detecting MRSA biofilm formation was conducted according to the method reported by Qian Chungu et al. Briefly, log-phase *MRSA* (2 × 10^7^ CFU/mL) was inoculated at 100 µL per well into a 96-well plate containing 100 µL/well of emodin sample solutions at different concentrations (1× MIC and 2× MIC, respectively). The plate was then incubated at 37 °C for 24 h. After gentle washing, the biofilms were stained with 200 µL of 1% crystal violet for 15 min. Subsequently, the excess dye was discarded, and the plate was washed three times with distilled water. After air-drying at room temperature, 200 µL of ethanol was added to each well and allowed to stand for 20 min to dissolve the crystal violet. Finally, a microplate reader was used to measure the OD_570_ (Optical Density at 570 nm) values to analyze the biofilm formation of MRSA cells.

### 2.6. Determination of DNA and RNA in the Supernatant

MRSA cultures in the logarithmic growth phase were collected and resuspended in phosphate-buffered saline (PBS) to an optical density at 600 nm (OD_600_) of 1. The bacterial suspension was then treated with emodin at 1× and 2× the minimum inhibitory concentration (MIC) for 6 h. Bacteria treated with 0.1% ethanol served as the vehicle control. After treatment, the supernatant was filtered through a 0.22 μm membrane and analyzed using a NanoDrop 2000 spectrophotometer (Thermo Fisher Scientific, Waltham, MA, USA) to determine the release of DNA and RNA by measuring the absorbance at 260 nm, following the method of Spadari with slight modifications [20].

### 2.7. SEM Characterization of Emodin’s Effect on MRSA Cell Morphology

The morphological changes of Methicillin-resistant *Staphylococcus aureus* (MRSA) cells after emodin treatment were characterized by scanning electron microscopy (SEM), as previously described with modifications. Briefly, logarithmic-phase bacteria were resuspended in fresh medium to approximately 1 × 10^8^ CFU/mL. The bacterial suspension was then treated with emodin at 1× and 2× the minimum inhibitory concentration (MIC) and incubated at 37 °C for 6 h. After incubation, the cells were washed three times with phosphate-buffered saline (PBS) and fixed with 2.5% glutaraldehyde at 4 °C overnight. The fixed samples were dehydrated through a graded ethanol series (50%, 70%, 90%, and 100% twice), spending 15 min in each concentration. Subsequently, the samples were critically point-dried for 2 h. Finally, the dried samples were sputter-coated with gold and observed under a Hitachi SU8020 (Hitachi, Tokyo, Japan) scanning electron microscope.

### 2.8. Proteomics Experiment

Sample Preparation for Proteomics Analysis [21]: MRSA was treated with emodin at 1× the minimum inhibitory concentration (1× MIC) for 4 h. The bacterial cells were then collected by centrifugation, and the pellet was washed twice with ice-cold phosphate-buffered saline (PBS). Subsequently, protein lysis buffer was added to the pellets from both the treated and control groups. The cells were lysed by sonication and centrifuged. The protein concentration in the supernatant was determined using a BCA Protein Assay Kit(Beyotime Biotechnology Co., Ltd. Shanghai, China) to verify extraction efficiency. The extracted proteins were digested with trypsin, desalted, and lyophilized. The resulting peptides were reconstituted in 40 μL of 0.1% formic acid solution. Finally, the solution was centrifuged, and the supernatant was collected for mass spectrometry analysis. The entire experiment was performed in three independent replicates.

### 2.9. Verification of Action Pathway

Intracellular reactive oxygen species (ROS) levels were measured using the fluorescent probe CM-H_2_DCFDA [22]. Briefly, after treatment with emodin at the specified concentrations, bacterial cells were collected, washed, and stained with 2 μM CM-H_2_ DCFDA at 37 °C for 45 min. Bacteria treated with 0.1% DMSO served as the control. After staining, the cells were washed with Hank’s Balanced Salt Solution (HBSS) and resuspended in the same buffer. ROS production was then analyzed by flow cytometry and quantified based on the median fluorescence intensity (MFI).

### 2.10. Evaluation of the Synergistic Antibacterial Effects of Emodin with Antibiotics

#### 2.10.1. Evaluation of Emodin-Mediated Enhancement of Cephalexin and Amoxicillin Against MRSA

The synergistic antibacterial activity of emodin combined with either cephalexin or amoxicillin against MRSA was evaluated using the broth microdilution checkerboard method, following Clinical and Laboratory Standards Institute (CLSI, 2017) guidelines. Briefly, logarithmic-phase MRSA was adjusted to 2 × 10^6^ CFU/mL in sterile cation-adjusted Mueller-Hinton broth (CAMHB). In a sterile 96-well plate, 50 µL of two-fold serially diluted antibiotic (in CAMHB) was added vertically, followed by the horizontal addition of 50 µL of two-fold serially diluted emodin (in DMSO, final in-well concentration ≤ 1%). Finally, 100 µL of the bacterial suspension was added to each well, yielding a final inoculum of 5 × 10^5^ CFU/mL.

Controls included positive growth (bacteria + CAMHB + solvent), negative sterility (CAMHB only), and single-agent gradient wells. After 24 h static incubation at 37 °C, wells with no visible growth were identified visually, and the optical density at 600 nm (OD_600_) was measured. To confirm, 10 µL samples from “no growth” and borderline OD wells were spotted onto Mueller-Hinton agar plates for colony counting after 24 h.

The minimum bactericidal concentration (MBC) of the combination was defined as the lowest concentration achieving a ≥99.9% reduction in colony count versus the initial inoculum. Synergy was quantified by the fractional inhibitory concentration index (FICI):FICI = (MIC_A-combo/MIC_A-alone) + (MIC_B-combo/MIC_B-alone). An FICI ≤ 0.5 was interpreted as synergism

#### 2.10.2. Inhibition of MRSA Adhesion to HaCaT Cells by Emodin and Cephalexin

(1) Drug Preparation and Bacterial Treatment

A single MRSA colony was inoculated into 20 mL of sterile cation-adjusted Mueller-Hinton broth (CAMHB) and incubated at 37 °C with shaking at 180 rpm until the logarithmic growth phase was reached. Cephalexin and Emodin were co-dissolved in dimethyl sulfoxide (DMSO) at a mass ratio of 1:4 and then diluted with CAMHB to the desired working concentrations. After vortexing, 100 μL of the drug solution was aliquoted into 15 mL centrifuge tubes, with three technical replicates per concentration. The logarithmic-phase *MRSA* culture was adjusted with sterile CAMHB to a concentration of 1.0 × 10^8^ CFU/mL (OD_600_ = 0.2). A 1 mL aliquot of this bacterial suspension was added to 5 mL centrifuge tubes containing the drug solutions, achieving final concentrations of cephalexin (20 μg/mL) alone or in combination with Emodin (20 μg/mL cephalexin + 2 μg/mL Emodin). The bacterial concentration in all tubes was maintained at 1.0 × 10^8^ CFU/mL. A control group containing an equivalent concentration of DMSO was included. All samples were incubated at 37 °C with shaking at 180 rpm for 4 h.

(2) Bacterial Staining with CFDA-SE

Following the 4 h drug treatment, the bacterial suspensions were adjusted to a final volume of 5.0 mL with PBS. The suspensions were then centrifuged at 4000× *g* for 10 min, and the supernatant was discarded. The bacterial pellets were washed twice with PBS by repeating the centrifugation and resuspension steps. The washed pellets were resuspended in 1.0 mL of PBS and stained with CFDA-SE stock solution according to the manufacturer’s instructions (e.g., at a final concentration of X μM). The mixture was gently vortexed and incubated at 37 °C for 20 min in the dark. After incubation, the stained bacteria were pelleted by centrifugation (4000× *g*, 10 min) and washed three times with 1% FBS/HBSS. Finally, the bacteria were resuspended in 1 mL of 1% FBS/HBSS for the subsequent adhesion assay.

(3) HaCaT Cell Culture and MRSA Adhesion Assay

HaCaT cells were seeded in a 12-well plate at a density of 5 × 10^5^ cells per well. The cells were cultured in Dulbecco’s Modified Eagle Medium (DMEM) supplemented with 10% FBS and 1% penicillin-streptomycin at 37 °C in a 5% CO_2_ incubator for 24 h to reach confluence, followed by an additional 24 h maturation period. Prior to the assay, the cell monolayer was washed twice with 1% FBS/HBSS. The CFDA-SE-stained *MRSA* (pre-treated with drugs or DMSO as described above) were added to the cells at a multiplicity of infection (MOI) of 20:1. The plate was then incubated at 37 °C for 2 h to allow bacterial adhesion. After incubation, non-adherent bacteria were removed by gently washing the monolayer three times with 1% FBS/HBSS. The wells were preserved in 1% FBS/HBSS for immediate imaging. MRSA adhesion to HaCaT cells was observed and quantified using an integrated cell imager. Images were captured using the green fluorescence channel appropriate for CFDA-SE to record the changes in the number of adherent MRSA following the different drug treatments.

#### 2.10.3. Effect of the Emodin-Cephalexin Combination on MRSA-Infected HaCaT Cells

MRSA from single colonies was inoculated into 20 mL of sterile cation-adjusted Mueller-Hinton broth (CAMHB) and incubated at 37 °C with shaking at 180 rpm until the logarithmic growth phase was reached.

(1) Bacterial Staining

The logarithmic-phase MRSA culture was adjusted to an OD_600_ of 0.2 in PBS (final volume: 5 mL) and centrifuged at 4000× *g* for 10 min. The pellet was washed twice with PBS. The bacterial pellet was then resuspended in 1 mL of PBS and stained with CFDA-SE. After gentle vortexing and incubation at 37 °C for 20 min in the dark, the stained bacteria were pelleted by centrifugation and washed three times with 1% FBS/HBSS. Finally, the bacteria were resuspended in 1 mL of 1% FBS/HBSS to a concentration of 1.0 × 10^8^ CFU/mL for subsequent use.

(2) Cell Preparation and Infection Assay

HaCaT cells were cultured in DMEM supplemented with 10% FBS and 1% penicillin-streptomycin at 37 °C in a 5% CO_2_ incubator for two days. The cells were then trypsinized, washed twice with 1% FBS/HBSS, counted, and resuspended in 1% FBS/HBSS at a concentration of 5.0 × 10^5^ cells/mL.

Cephalexin and Emodin were co-dissolved in DMSO at a mass ratio of 1:4 and subsequently diluted in 1% FBS/HBSS to prepare working solutions.

Aliquots of 100 µL of the cell suspension (containing 5 × 10^4^ cells) were transferred to 2 mL centrifuge tubes. To each tube, 100 µL of the respective drug solution was added, achieving final concentrations of 20 µg/mL cephalexin alone or 20 µg/mL cephalexin combined with 2 µg/mL Emodin. Each treatment was performed in triplicate. A control group receiving an equivalent volume of DMSO was included.

CFDA-SE-stained MRSA was then added to each tube at a multiplicity of infection (MOI) of 20:1. The final bacterial concentration was 1.0 × 10^7^ CFU/mL, and the final cell concentration was 2.5 × 10^5^ cells/mL. The samples were incubated at 37 °C with gentle shaking (60 rpm) for 2 h.

(3) Flow Cytometry Analysis

After incubation, the samples were centrifuged at 1000× *g* for 5 min to pellet the cells. This washing step was repeated twice with 1% FBS/HBSS to remove unbound bacteria. The final cell pellet was resuspended in 200 µL of 1% FBS/HBSS for flow cytometric analysis.

Samples were analyzed using a flow cytometer with the FL-1 channel. The photomultiplier tube voltage was first adjusted using uninfected HaCaT cells to set the baseline fluorescence. Subsequently, the MRSA-infected samples were analyzed, and data for at least 10,000 events per tube were collected.

#### 2.10.4. Evaluation of the Effects of Emodin-Amoxicillin Combination on MRSA-Infected RAW264.7 Cells

(1) Drug Treatment and Fluorescent Labeling of *MRSA*

Log-phase MRSA was resuspended in fresh CAMHB to an OD_600_ of 0.2 and then treated with the following drugs at 37 °C with shaking for 4 h: amoxicillin (10 μg/mL), or a combination of amoxicillin and Emodin (10 μg/mL + 2 μg/mL). A control group was treated with 0.1% DMSO. After incubation, the bacterial cells were collected by centrifugation at 4 °C and washed twice with ice-cold PBS. The bacterial pellets were resuspended in 1 mL of PBS and stained with CFDA-SE at a final concentration of 2 μM. The suspensions were gently vortexed and incubated at 37 °C for 20 min in the dark. Subsequently, the stained bacteria were collected by centrifugation, washed three times with 1% FBS/HBSS, and finally resuspended in 1 mL of 1% FBS/HBSS. The labeled bacteria were used within 2 h.

(2) Infection of RAW264.7 Cells and Flow Cytometric Analysis

RAW264.7 cells were seeded in a 6-well plate at a density of 5×10^5^ cells per well and incubated for 12 h at 37 °C in a 5% CO_2_ atmosphere to allow adherence. The culture medium was aspirated, and the cell monolayer was gently washed once with 1% FBS/HBSS. The cells were then incubated with the CFDA-SE-labeled *MRSA* suspension at a multiplicity of infection (MOI) of 2.5:1 for 2 h at 37 °C. After the infection period, the supernatant was removed. The cells were washed twice with 1% FBS/HBSS to remove non-adherent bacteria and subsequently detached from the plate using a cell scraper. The cell suspension was collected by centrifugation and washed twice more with 1% FBS/HBSS. The final cell pellet was resuspended in flow cytometry buffer. Samples were analyzed using a flow cytometer. The population of intact cells was gated based on forward and side scatter characteristics. CFDA-SE fluorescence was detected in the FL-1 channel. The infection rate was determined by the shift in fluorescence intensity of the infected samples compared to the autofluorescence of uninfected RAW264.7 cells. Data from at least 10,000 events within the cell gate were collected for each sample.

## 3. Results

### 3.1. Screening of Active Components

To identify the active components in *R. officinale* against Methicillin-resistant *Staphylococcus aureus* (MRSA), the antibacterial activity of four compounds isolated from rhubarb was evaluated using the broth microdilution assay. The minimum inhibitory concentrations (MICs) of the compounds against MRSA were determined as follows: emodin, 12 μg/mL; rhein, 16 μg/mL; aloe-emodin, >100 μg/mL; and chrysophanol, >100 μg/mL. These results indicate that emodin and rhein are the key active substances contributing to rhubarb’s anti-MRSA activity.

### 3.2. Antimicrobial Susceptibility Test

As shown in Table 1 and Table 2, emodin exhibited the lowest minimum inhibitory concentration (MIC) against MRSA among the compounds tested, including rhein and the conventional antibiotics amoxicillin and cephalexin. This indicates that emodin possesses superior in vitro anti-MRSA activity. In contrast, aloe-emodin, chrysophanol, berberine, flavonol, and baicalin showed no appreciable activity at the concentrations tested.

### 3.3. Emodin Inhibits the Growth of MRSA

**Figure 1:**
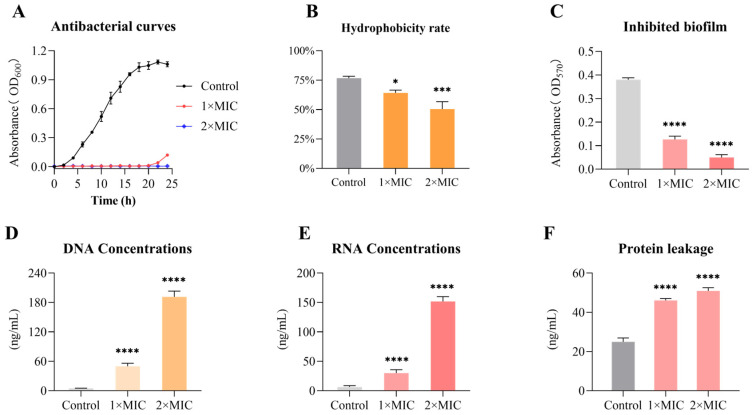
Phenotypic effects of emodin on MRSA. (**A**) Growth curves of MRSA treated with emodin. Bacterial growth (OD_600_) was monitored over time in the presence of emodin at 1× MIC, 2× MIC, or a vehicle control. Vancomycin was used as a positive control. (**B**) Effect of emodin on cell surface hydrophobicity. The hydrophobicity of MRSA cells after emodin treatment was assessed using the microbial adhesion to hydrocarbon (MATH) assay. (**C**) Inhibitory effect of emodin on biofilm formation. Biofilms formed by MRSA after 24 h treatment with emodin were quantified by crystal violet staining (OD_570_). (**D**,**E**) Emodin induces nucleic acid leakage. The release of nucleic acids into the supernatant from emodin-treated *MRSA* was quantified by measuring the absorbance at (**D**) 260 nm and (**E**) 230 nm. (**F**) Emodin induces protein leakage. Protein concentration in the supernatant of emodin-treated *MRSA* was determined using a BCA assay.(* *p* < 0.05 *** *p* < 0.001, **** *p* < 0.0001 vs. control).

The effect of emodin on *MRSA* growth was determined by time-kill kinetics. Exponential-phase MRSA was treated with emodin, and bacterial growth was monitored by measuring the optical density at 600 nm (OD_600_) over time. Vancomycin (MIC = 1 μg/mL) served as a positive control. As shown in Figure 1A, the OD_600_ of the control group began to increase after 2 h, indicating normal growth. In the presence of 1× MIC emodin, growth was suppressed for 20 h before a gradual increase in OD_600_ was observed. In contrast, treatment with 2× MIC emodin completely inhibited bacterial growth throughout the experiment. These results demonstrate that emodin inhibits MRSA proliferation in a dose-dependent manner.

### 3.4. Emodin Decreases the Cell Surface Hydrophobicity of MRSA

The inhibitory effect of emodin on methicillin-resistant *Staphylococcus aureus* (MRSA) biofilm formation was assessed using the crystal violet staining assay, in line with established microbiological protocols. MRSA strains in the exponential growth phase were exposed to emodin at distinct concentrations (1× MIC and 2× MIC) for 24 h, followed by crystal violet staining. The level of biofilm formation was then quantitatively determined by measuring absorbance at 570 nm (OD_570_).

As illustrated in Figure 1C, emodin exerted a significant, dose-dependent inhibitory effect on MRSA biofilm formation. At 1× MIC, biofilm biomass was reduced by over 70% relative to the untreated control group. When the emodin concentration was escalated to 2× MIC, the OD_570_ value declined to near-baseline levels (OD_570_ < 0.1), which was comparable to that of the blank control group. Collectively, these data demonstrate that emodin at 2× MIC completely abrogates MRSA biofilm formation; following 24 h of treatment at this concentration, MRSA was unable to generate detectable biofilm structures.

Emodin exerts dose-dependent effects in both inhibiting the initiation of MRSA biofilm formation and disrupting pre-established MRSA biofilms [23], a phenomenon indicative of its multi-stage anti-biofilm activity across the biofilm lifecycle. The present study specifically focuses on the formative phase of biofilm development, providing definitive evidence that emodin potently and dose-dependently suppresses MRSA biofilm establishment at this critical early stage. Collectively, these complementary findings consolidate the core mechanistic trait of emodin’s dose-dependent anti-MRSA biofilm activity, underscoring its potential as a broad-spectrum biofilm-targeting agent.

### 3.5. Inhibitory Effect of Emodin on MRSA Biofilm Formation

Biofilm formation was assessed using the crystal violet staining method. Exponential-phase MRSA was treated with emodin at sub-inhibitory (1× MIC) and inhibitory (2× MIC) concentrations for 24 h. After staining, the biomass of the formed biofilms was quantified by measuring the optical density at 570 nm (OD_570_). As shown in Figure 1C, emodin treatment significantly reduced the OD_570_ value—a proxy for biofilm biomass—in a dose-dependent manner. At 1× MIC, emodin inhibited biofilm formation by more than 70%. At 2× MIC, the OD_570_ decreased to a level below 0.1, which was comparable to the negative (sterile medium) control. This result indicates that emodin, at a concentration of 2× MIC, almost completely prevented the formation of a detectable biofilm by MRSA under these experimental conditions.

### 3.6. Emodin Induces Nucleic Acid Leakage in MRSA

The integrity of the MRSA cell membrane after emodin treatment was evaluated by measuring the leakage of nucleic acids into the supernatant. Bacterial cultures were treated with emodin at 1× MIC and 2× MIC for 6 h at 37 °C, after which the absorbance of the cell-free supernatant at 260 nm (A_260_)—which indicates the presence of nucleic acids—was quantified. As shown in Figure 1D, the A_260_ value in the supernatant of the 1× MIC treatment group was higher than that of the control group. This value was significantly further increased in the 2× MIC treatment group, demonstrating a dose-dependent effect of emodin on nucleic acid release. A parallel measurement at 230 nm (A_230_) showed a similar trend (Figure 1E), corroborating the findings. Collectively, these results indicate that emodin compromises the cell membrane integrity of *MRSA*, leading to the leakage of intracellular nucleic acids.

### 3.7. Emodin Alters the Morphology of MRSA

**Figure 2:**
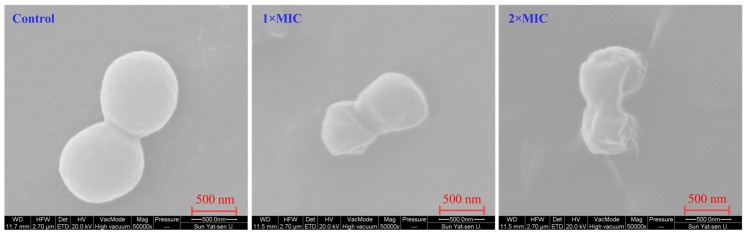
Emodin induces morphological alterations in *MRSA*. Representative scanning electron microscopy (SEM) images of *MRSA* cells following a 6 h treatment with emodin at its 1× MIC (12 μg/mL) and 2× MIC (24 μg/mL). Untreated cells and cells treated with the vehicle control (0.1% DMSO) display smooth and intact surfaces. In contrast, emodin treatment resulted in severe, concentration-dependent deformation, including cell surface wrinkling, collapse, and rupture.

The morphological changes in MRSA following emodin treatment were examined by scanning electron microscopy (SEM) (Figure 2). Untreated control cells displayed smooth and intact surfaces. In contrast, emodin treatment induced significant concentration-dependent morphological alterations. At 1× MIC, bacterial surfaces appeared wrinkled and shrunken. Treatment with 2× MIC emodin resulted in severe surface collapse and cellular deformation in the majority of cells. These morphological disruptions are consistent with the observed leakage of intracellular nucleic acids and proteins, indicating that emodin compromises the integrity of the bacterial cell envelope.

### 3.8. Emodin Exerts Antibacterial Effects Through Systemic Proteomic Alterations in MRSA

**Figure 3:**
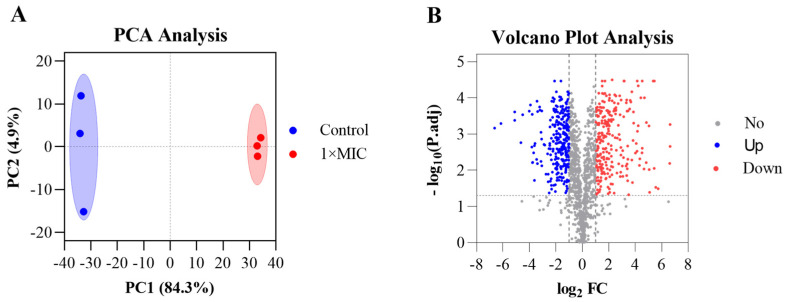
Proteomic profiling of MRSA in response to emodin treatment. (**A**) Principal component analysis (PCA) plot showing clear separation between the control and emodin-treated (1×MIC) groups along the first principal component (PC1). (**B**) Volcano plot displaying differentially expressed proteins (DEPs) between control and emodin-treated samples. Proteins with a significant change (|Fold change| > 1.5 and *p* < 0.05) are highlighted in red (upregulated) and blue (downregulated). Grey dots represent non-significant proteins.

Principal Component Analysis (PCA) (Figure 3A): PCA revealed a clear separation between the emodin-treated and control groups, with the first principal component (PC1) accounting for 84.3% of the total variance. This distinct clustering indicates that emodin treatment is the predominant factor driving the observed differences and has induced substantial, consistent, and global changes in the proteomic profile, ruling out minor stochastic variations. Differentially Expressed Proteins (DEPs) (Figure 3B): Volcano plot analysis identified 485 DEPs, with a comparable number of proteins being upregulated (*n* = 238) and downregulated (*n* = 247). The balanced distribution between up- and down-regulation demonstrates that emodin’s mechanism is not a unilateral suppression but involves a complex, multi-targeted regulatory response. This pattern suggests a simultaneous disruption of essential biological pathways and an activation of bacterial stress response systems.The proteomic data robustly demonstrate that emodin exerts its antibacterial activity against MRSA through a systematic, multi-faceted mechanism rather than via a single target. By broadly perturbing the bacterial protein expression network, emodin likely compromises cellular integrity, inhibits core metabolic functions, and overwhelms adaptive stress responses concurrently. This multi-target nature is particularly advantageous as it may significantly reduce the likelihood of drug resistance development in bacteria.

### 3.9. Emodin Perturbs Key Physiological Processes in MRSA

#### 3.9.1. Suppression of Antioxidant and Virulence Proteins

**Figure 4:**
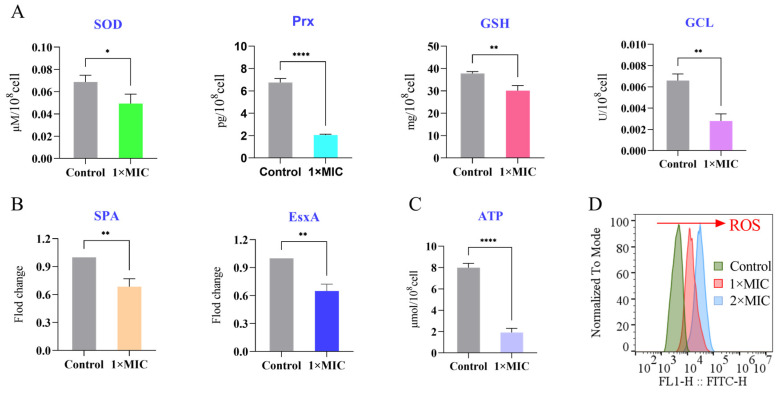
Emodin disrupts key physiological functions in MRSA. MRSA was treated with emodin at 1× MIC and 2× MIC for 4 h. (**A**) Intracellular antioxidant capacity was significantly reduced, as determined by a [Specify, e.g., glutathione] assay. (**B**) The expression of key virulence factors (SpA and EsxA) was downregulated. (**C**) Intracellular ATP levels were depleted in a concentration-dependent manner. (**D**) Intracellular ROS levels were significantly elevated, as measured by flow cytometry using CM-H_2_DCFDA staining.(* *p* < 0.05,** *p* < 0.01 **** *p* < 0.0001 vs. control).

Proteomic analysis revealed that emodin treatment significantly downregulated the expression of key proteins involved in oxidative stress defense and virulence in MRSA (Figure 4A). Notably, the abundance of central antioxidant enzymes, including superoxide dismutase (SOD), peroxiredoxin (Prx), and glutamate-cysteine ligase (GCL), was reduced. Concurrently, the expression of major virulence factors, Staphylococcal protein A (SpA) and ESAT-6-like protein EsxA, was also markedly decreased.

#### 3.9.2. Depletion of Intracellular ATP

The effect of emodin on bacterial energy status was assessed by measuring intracellular ATP levels. As shown in Figure 4C, treatment with emodin at its MIC resulted in a 73.8% reduction in ATP concentration compared to the control group, indicating a severe impairment of cellular energy metabolism.

#### 3.9.3. Induction of Reactive Oxygen Species (ROS)

Intracellular ROS levels were quantified using CM-H_2_ DCFDA staining. Flow cytometric analysis demonstrated that emodin treatment triggered a significant accumulation of ROS in MRSA cells compared to the control (Figure 4D).

### 3.10. Emodin Synergizes with β-Lactam Antibiotics Against MRSA

**Figure 5:**
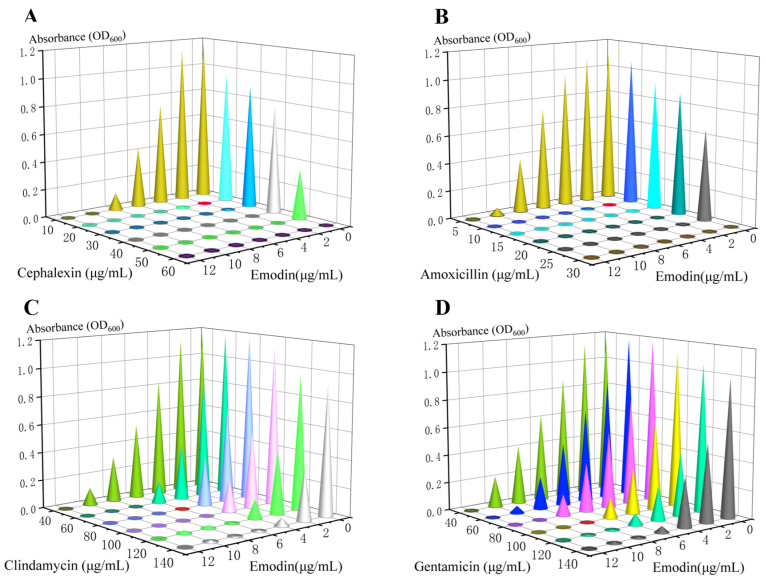
Emodin synergizes with antibiotics to inhibit MRSA growth. Growth curves of *MRSA* treated with emodin in combination with (**A**) cephalexin, (**B**) amoxicillin, (**C**) clindamycin, or (**D**) gentamicin for 24 h. Bacterial growth was measured as the optical density at 600 nm (OD_600_). The enhanced inhibition in the combination therapy, compared to each agent alone, demonstrates a synergistic effect.

The potential synergy between emodin and conventional antibiotics was evaluated using the checkerboard assay and quantified by the fractional inhibitory concentration index (FICI). As shown in Figure 5, the combination of emodin with cephalexin or amoxicillin resulted in enhanced inhibition of *MRSA* growth compared to each agent alone. The FICI for the emodin-cephalexin combination was 0.475, and for the emodin-amoxicillin combination, it was 0.5 (Table 3). As both FICI values are below the 0.5 threshold, these results demonstrate a synergistic interaction between emodin and these two β-lactam antibiotics against MRSA.

For antibiotics where the measured MIC exceeded the highest tested concentration (200 μg/mL for clindamycin and gentamicin), a value of 200 μg/mL was used for FICI calculations. The resulting FICI values were interpreted as follows: ≤0.5, synergy; >0.5 to <1, additive; =1, indifferent.

### 3.11. Synergistic Antibacterial Composition Inhibits the Adhesion of MRSA to HaCaT Cells

**Figure 6:**
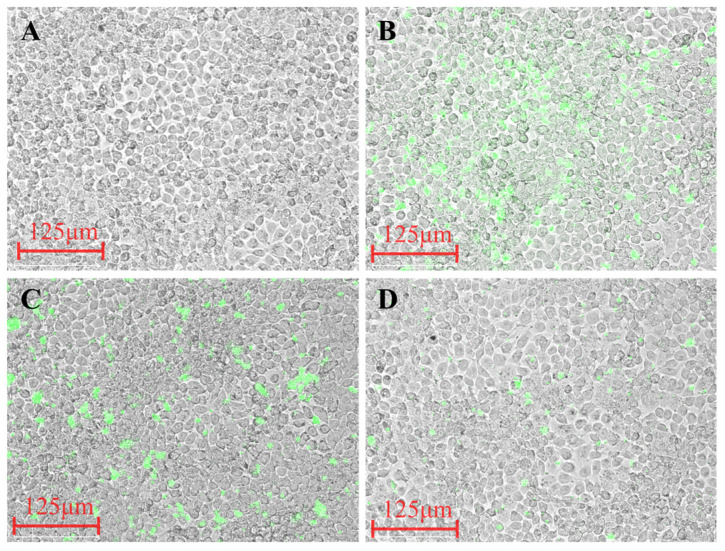
The Cephalexin and Emodin combination inhibits *MRSA* adhesion to HaCaT cells. HaCaT cells were infected with CFSE-labeled MRSA (green) at an MOI of 10:1 for 2 h after the bacteria were pre-treated for 4 h with the specified agents. Non-adherent bacteria were removed by washing prior to fluorescence imaging. Representative images show: (**A**) Blank control (sterile medium), (**B**) Vehicle control (DMSO), (**C**) Cephalexin (20 μg/mL) alone, and (**D**) Cephalexin (20 μg/mL) in combination with Emodin (2 μg/mL). A substantial reduction in adherent *MRSA* is observed in the combination therapy group (**D**). Scale bar = 20 μm.

The adhesion of MRSA to HaCaT cells was evaluated following treatment of the bacteria with different drug regimens. As shown in Figure 6, CFDA-SE-labeled *MRSA* (green) adhering to the cell monolayer was visualized using fluorescence microscopy. Comparison of the treatment groups revealed a substantial reduction in adherent bacteria in the Cephalexin + Emodin combination group (Figure 6D) compared to the untreated control (Figure 6B), Cephalexin alone (Figure 6C), or other groups. This demonstrates that the combination of Cephalexin and Emodin significantly inhibits the adhesion of MRSA to host epithelial cells.

### 3.12. The Cephalexin and Emodin Combination Synergistically Inhibits MRSA Invasion of HaCaT Cells

**Figure 7:**
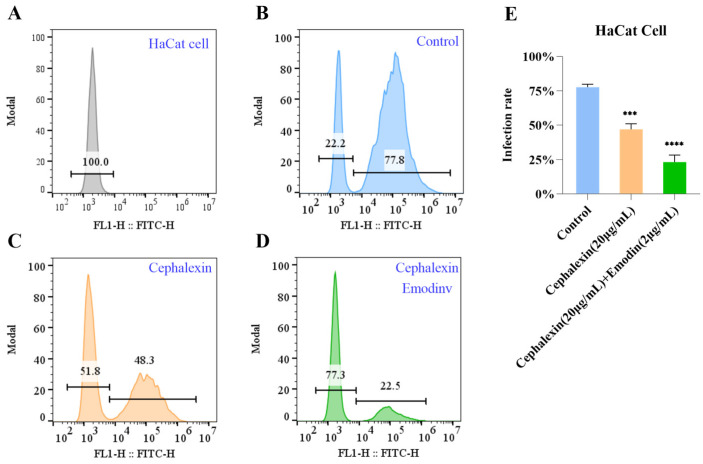
Emodin enhances the efficacy of cephalexin in inhibiting MRSA infection of HaCaT cells. Panel (**A**): Gating strategy for HaCaT cells by flow cytometry, establishing the baseline for distinguishing infected and uninfected cell populations. Panel (**B**): Flow cytometric analysis of the vehicle control group, revealing a high baseline infection rate of 77.8%. Panel (**C**): Treatment with cephalexin (20 μg/mL) significantly reduced the proportion of infection-positive cells to 48.3%. Panel (**D**): The combination of cephalexin (20 μg/mL) and emodin (2 μg/mL) resulted in a more pronounced reduction, with only 22.3% of cells being infection-positive. Panel (**E**): Quantitative summary of infection rates across treatment groups. Data are presented as mean ± SD (n = 3). Statistical significance was determined by one-way ANOVA (*** *p* < 0.001, **** *p* < 0.0001 vs. control; *p* < 0.001 vs. cephalexin alone).

The efficacy of the drug treatments in preventing MRSA invasion into HaCaT cells was quantified by flow cytometry, measuring the proportion of infection-positive cells. As shown in Figure 7B–D and the corresponding statistical summary, the infection rate was profoundly affected by the treatments. In the control group, 77.8% of the cells were infected. This rate was significantly reduced to 48.3% by treatment with cephalexin (20 μg/mL) alone (*p* < 0.001). Most notably, the combination of cephalexin (20 μg/mL) and emodin (2 μg/mL) exhibited a synergistic anti-infective effect, further suppressing the infection rate to 22.3% (*p* < 0.0001 compared to the control). These results clearly demonstrate that the cephalexin-emodin combination is highly effective in blocking *MRSA* internalization into host cells.

### 3.13. Emodin Enhances the Efficacy of Amoxicillin Against MRSA Infection in RAW 264.7 Cells

**Figure 8:**
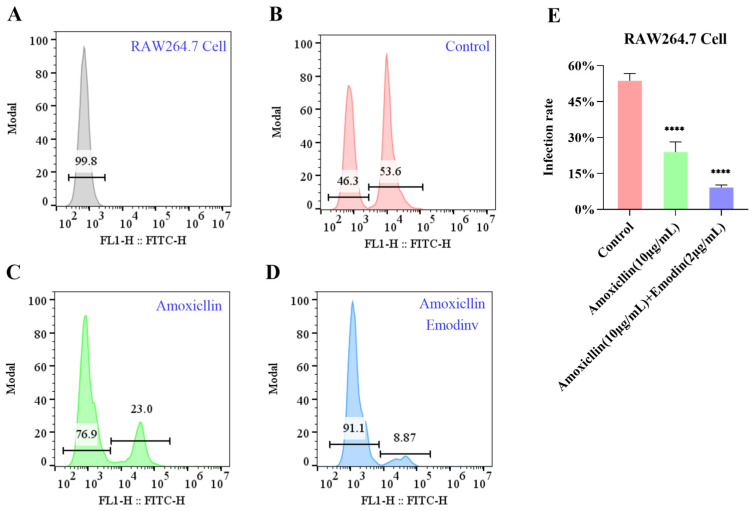
Emodin enhances the efficacy of amoxicillin against MRSA infection in RAW 264.7 macrophages. Panel (**A**): Gating strategy for RAW 264.7 macrophages by flow cytometry, establishing the baseline for distinguishing infected and uninfected cell populations. Panel (**B**): Flow cytometric analysis of the control group, showing a baseline infection rate of 53.6%. Panel (**C**): Treatment with amoxicillin significantly reduced the proportion of infection-positive cells to 23.0%. Panel (**D**): The combination of amoxicillin and emodin resulted in a more pronounced reduction, with only 8.87% of cells being infection-positive. Panel (**E**): Quantitative summary of infection rates. Data are presented as mean ± SD (n = 3). Statistical significance was determined by one-way ANOVA (**** *p* < 0.0001 vs. control).

The efficacy of amoxicillin, alone and in combination with emodin, in preventing *MRSA* infection of RAW 264.7 macrophages was quantified by flow cytometry. As shown in Figure 8B–E, both treatments significantly reduced the percentage of infection-positive cells. The baseline infection rate in the control group was 53.6%. Treatment with amoxicillin alone significantly reduced this rate to 23.0% (*p* < 0.0001). The combination of amoxicillin and emodin exhibited a superior anti-infective effect, further suppressing the infection rate to 8.87% (*p* < 0.0001). These results demonstrate that emodin potently enhances the ability of amoxicillin to block MRSA infection in macrophages.

## 4. Discussion

Confronting the increasingly severe global crisis of antimicrobial resistance (AMR), particularly the clinical challenges posed by methicillin-resistant Staphylococcus aureus (MRSA), there is an urgent and unmet need to develop novel therapeutic strategies. This study demonstrates that emodin—the primary anthraquinone phytoconstituent of Rheum officinale (rhubarb)—exerts potent anti-MRSA activity via a multi-target mechanism of action and functions as a highly effective synergistic adjuvant for β-lactam antibiotics. Below, we contextualize the core findings of this work within the broader scientific landscape, systematically dissecting their congruence with and divergence from prior investigations to highlight the study’s incremental contributions to the field.

### 4.1. Emodin as a Multi-Target Anti-MRSA Agent

(1) Deepening and Integrating Known Mechanisms

A central conclusion of this study is that emodin compromises MRSA viability by simultaneously disrupting multiple cellular structures and homeostatic functions. This multi-target pharmacology is hypothesized to significantly delay the emergence of adaptive resistance [24], which aligns with the well-established paradigm that natural antimicrobials often exhibit polypharmacological effects to circumvent single-target resistance evolution [25]. Unlike conventional single-target antibiotics, highly efficacious natural antimicrobials typically deploy a “multi-pronged” mode of action, interfering with parallel bacterial physiological pathways to mitigate rapid resistance acquisition [26]. However, the novelty of this study lies not only in validating emodin’s multi-target potential but, more critically, in deepening and integrating mechanistic insights into the specific functions of these targets and their interdependencies via rigorous, systematic experimental evidence—surpassing the relatively isolated or phenomenological mechanistic descriptions in prior literature.

(2) Deepening Congruence in Bacterial Membrane Damage

This study confirms that emodin induces severe perturbation of the MRSA cell envelope, with dose-dependent nucleic acid leakage and ultrastructural morphological damage visualized via SEM (Figure 1D,E and Figure 2) providing direct, corroborative evidence of membrane disruption. This finding is highly consistent with prior reports demonstrating emodin’s capacity to interact with and destabilize bacterial membranes [27]. The key differential contribution of our work is that, rather than merely recapitulating this phenomenon, we establish a direct causal link between membrane perturbation and ultimate bacterial cell death through dose–response profiling and correlative functional assays, thereby reinforcing the assertion that membrane damage constitutes a core lethal mechanism of emodin action. Concurrently, the observed reduction in MRSA cell surface hydrophobicity (Figure 1B) extends current understanding of emodin’s impact on membrane and surface biophysics, furnishing novel mechanistic clues for its inhibition of biofilm formation—a critical virulence determinant [28]—and representing a substantive advancement over prior membrane-focused investigations.

(3) Systematic Expansion of Metabolic and Oxidative Stress Mechanisms

By integrating label-free proteomics with targeted functional assays, this study unravels profound emodin-induced metabolic dysregulation (Figure 3 A,B), intracellular ATP depletion (Figure 4C), and exacerbation of endogenous oxidative stress (Figure 4A,D). These findings—particularly the explicit linkage and quantitative characterization of metabolic collapse and oxidative stress cascades—mark a critical mechanistic deepening relative to earlier studies. While metabolic interference and oxidative stress are well-documented in natural product-mediated antimicrobial activity, our work clearly delineates the specific molecular manifestations of these processes under emodin exposure (e.g., downregulation of core glycolytic and tricarboxylic acid cycle proteins, depletion of key antioxidant enzymes) and their non-additive, synergistic lethal effects [29,30]. The conceptualization of membrane damage, metabolic paralysis, and oxidative injury as an interconnected “vicious cycle” or synergistic network establishes a more holistic and mechanistically complete model, representing a paradigm shift in the understanding of emodin’s antibacterial pharmacology.

(4) Explicit Revelation of Anti-Virulence Activity

This study provides definitive evidence that emodin downregulates the expression of MRSA’s key virulence factors SpA and EsxA (Figure 4A), thereby expanding its functional repertoire to encompass “anti-virulence” activity [31]. This discovery constitutes a marked divergence from, and critical extension of, prior studies that focused exclusively on emodin’s direct bactericidal or bacteriostatic activity. It shifts emodin’s mode of action from the traditional bactericidal “kill” paradigm to the clinically promising “disarmament” strategy [32], adding a novel dimension to its multi-target profile and providing a theoretical framework for its potential to exert reduced resistance selective pressure—a key advantage over conventional antibiotics in combating AMR.

### 4.2. Synergy with β-Lactam Antibiotics: Advancing from Mechanistic Validation to Functional Translational Insights

Our study identifies robust synergistic interactions between emodin and β-lactam antibiotics (cephalexin, amoxicillin) (FICI < 0.5, Table 2) and proposes a “weaken-and-attack” synergistic model predicated on membrane damage and proton motive force (PMF) dissipation [33]. In broad terms, this finding aligns with the established research paradigm of natural products serving as antibiotic adjuvants to restore efficacy against drug-resistant pathogens [34]. However, the study’s prominent incremental contributions are twofold:

Targeted resolution of MRSA’s PBP2a-mediated resistance: We explicitly link the observed synergy to the circumvention of MRSA’s unique PBP2a-driven β-lactam resistance [33], grounding the “weaken-and-attack” model in a clinically relevant, pathogen-specific mechanism and moving beyond generic synergistic frameworks.

Validation of functional in vitro translational endpoints: Beyond standard in vitro pharmacodynamic validation, we further demonstrate in physiologically relevant host–cell models (HaCaT keratinocytes and murine macrophages) that this chemical synergy translates into tangible biological benefits—including reduced bacterial adhesion and invasion, and enhanced host-mediated immune clearance (Figure 6, Figure 7 and Figure 8) [35]. This integration of chemical synergy with host–pathogen interaction endpoints significantly elevates the translational credibility of our findings, bridging the gap between in vitro efficacy and potential clinical utility.

### 4.3. Considerations for Emodin’s Photosensitizer Properties

Emerging literature indicates that emodin’s antibacterial activity may be partially mediated via light-dependent reactive oxygen species (ROS)-generating pathways [14]. While this pathway was not incorporated into the core experimental framework of the current study, this unique photophysical property offers critical directional guidance and experimental implications for future investigations [36]:

Standardization of experimental photic conditions: Future work must rigorously control for light exposure (e.g., implementation of validated light-shielded protocols or definition of standardized illumination parameters) to disentangle the contributions of emodin’s intrinsic biochemical activity from its potential photodynamic effects, ensuring mechanistic clarity [37].

Exploitation of photodynamic enhancement: A high-priority future direction is the systematic quantification of photodynamic enhancement by comparing antimicrobial efficacy, ROS production, and biofilm inhibition profiles of emodin (alone and in antibiotic combinations) under dark conditions versus defined wavelength illumination [38].

Development of localized photodynamic therapies: Emodin’s photosensitizing properties open new avenues for the design of targeted, localized photodynamic antimicrobial therapies, where spatiotemporal control of illumination could augment antibacterial efficacy while minimizing systemic exposure and off-target toxicity—addressing a critical unmet need in topical MRSA infection management.

### 4.4. Implications and Future Perspectives: Mechanism-Driven Research Priorities

In summary, this study advances the understanding of emodin’s anti-MRSA activity across multiple mechanistic tiers: it confirms and refines the canonical membrane damage pathway, systematically integrates metabolic collapse, oxidative stress, and anti-virulence into a unified mechanistic network, and validates its translational potential as a β-lactam antibiotic adjuvant. Collectively, these findings establish emodin as a dual-potential lead compound—both a multi-target anti-MRSA agent and a resistance-mitigating antibiotic adjuvant.

Building on these mechanistic insights, the proposed future research agenda is uniquely targeted to address current limitations and accelerate translational progress:

(1) In vivo efficacy and safety validation in MRSA infection animal models to confirm in vitro findings and establish therapeutic windows [39];
(2) High-resolution target deconvolution using advanced techniques to identify direct molecular targets of emodin and refine its multi-target pharmacology [40];
(3) Formulation optimization to overcome emodin’s poor aqueous solubility and bioavailability, via nanocarrier or prodrug strategies, to enable clinical development [41].

These priorities are designed to translate the mechanistic advances reported here into tangible therapeutic solutions for combating MRSA and broader AMR threats.

## 5. Conclusions

This study demonstrates that emodin exerts potent anti-MRSA activity through a multi-target mechanism and exhibits significant synergy with β-lactam antibiotics.

Direct antibacterial and anti-biofilm effects: Emodin inhibited MRSA growth in a dose-dependent manner and substantially suppressed biofilm formation at 2× MIC. It compromised cell membrane integrity, as evidenced by morphological damage and the leakage of nucleic acids.

Underlying mechanisms of action: Proteomic and functional analyses revealed that emodin disrupts critical cellular processes in *MRSA*. It induces oxidative stress by inhibiting key antioxidant enzymes, attenuates virulence by downregulating SpA and EsxA, and impairs central metabolism—particularly carbohydrate and amino acid metabolism—thereby disrupting energy homeostasis and biosynthesis.

Synergistic potential with antibiotics: Emodin synergized with cephalexin and amoxicillin (FICI < 0.5). The emodin-cephalexin combination potently inhibited the adhesion and invasion of *MRSA* to HaCaT keratinocytes, while the emodin-amoxicillin combination enhanced the clearance of *MRSA* infection in RAW 264.7 macrophages.

In summary, this work elucidates the multi-faceted anti-*MRSA* mechanism of emodin and validates its potential as a synergistic adjuvant to β-lactam antibiotics. This strategy offers a promising approach to augment conventional antibiotics, potentially lowering required dosages and circumventing resistance in clinical practice.

## Figures and Tables

**Table 1:** Minimum inhibitory concentrations (MICs) of various compounds against *Staphylococcus aureus* (MRSA ATCC 43300).

Samples	MIC (μg/mL)
Emodin	12.0
Rhein	16.0
Aloe-emodin	>100.0
Chrysophanol	>100.0
Berberine	>100.0
Flavonols	>200 μg/mL
Baicalin	>200 μg/mL

**Table 2:** Minimum inhibitory concentrations (MICs) of Multiple antibiotics against *Staphylococcus aureus* (MRSA ATCC 43300).

Antibiotics	MIC (μg/mL)
Amoxicillin	30.0
Cephalexin	65.0
Gentamicin Sulfate	>200.0
Clarithromycin	>200.0
Clindamycin Hydrochloride	>200.0
Tetracycline	200.0
Vancomycin Hydrochloride	3.0

**Table 3:** Fractional inhibitory concentration index (FICI) values for the combination of emodin with antibiotics against *Staphylococcus aureus* (MRSA ATCC 35984).

Antibiotics	MIC (μg/mL)	FICI
Emodin	12.0	--
Cephalexin	65	--
Amoxicillin	30	--
Emodin + Cephalexin	2.0 + 20	0.475
Emodin + Amoxicillin	2.0 + 10	0.5
Emodin + Clindamycin	6.0 + 80	0.9
Emodin + Gentamicin	8.0 + 100	1.16

## Data Availability

The original contributions presented in this study are included in the article. Further inquiries can be directed to the corresponding author.

## Abbreviations

The following abbreviations are used in this manuscript:

*MRSA*	Methicillin-resistant Staphylococcus aureus
TCM	Traditional Chinese medicine
ROS	Reactive oxygen species
MHB	Mueller Hinton Broth
ATP	Adenosine triphosphate
CFU	colony forming units
CLSI	Clinical and Laboratory Standards Institute
SEM	scanning electron microscopy
MBC	minimal bactericidal concentration
MIC	minimum inhibitory concentration
NIST	National Institute of Standards and Technology
SDH	succinate dehydrogenase
NADH	nicotinamide adenine dinucleotide
OD	optical density
DNA	Deoxyribonucleic acid
RNA	Ribonucleic Acid
PCA	principal component analysis
FC	fold change
p.adj	p-value adjusted
SpA	Staphylococcal protein A
FICI	fractional inhibitory concentration index
DEPs	Differentially Expressed Proteins
EsxA	ESAT-6-like protein
CFDA-SE	Carboxy fluorescein Diacetate Succinimidyl Ester
DMEM	Dulbecco’s Modified Eagle Medium
MOI	multiplicity of infection
CAMHB	Cation-Adjusted Mueller-Hinton Broth
PBS	phosphate-buffered saline
SOD	Superoxide Dismutase
Prx	Peroxiredoxin
GCL	Glutamate-Cysteine Ligase
